# Therapeutic effects and mechanism of Atractylodis rhizoma in acute lung injury: Investigation based on an Integrated approach

**DOI:** 10.3389/fphar.2023.1181951

**Published:** 2023-04-24

**Authors:** Kun Shi, Yan Wang, Yangxin Xiao, Jiyuan Tu, Zhongshi Zhou, Guosheng Cao, Yanju Liu

**Affiliations:** ^1^ College of Pharmacy, Hubei University of Chinese Medicine, Wuhan, China; ^2^ Center for Hubei TCM Processing Technology Engineering, Wuhan, China

**Keywords:** acute lung injury, ethanolic extract of Atractylodis rhizoma, network pharmacology, metabolomics, molecular mechanisms

## Abstract

Acute lung injury (ALI) is characterized by an excessive inflammatory response. *Atractylodes lancea* (Thunb.) DC. is a traditional chinese medicine with good anti-inflammatory activity that is commonly used clinically for the treatment of lung diseases in China; however, its mechanism of against ALI is unclear. We clarified the therapeutic effects of ethanol extract of Atractylodis rhizoma (EEAR) on lipopolysaccharide (LPS)-induced ALI by evaluation of hematoxylin-eosin (HE) stained sections, the lung wet/dry (W/D) ratio, and levels of inflammatory factors as indicators. We then characterized the chemical composition of EEAR by ultra-performance liquid chromatography and mass spectrometry (UPLC-MS) and screened the components and targets by network pharmacology to clarify the signaling pathways involved in the therapeutic effects of EEAR on ALI, and the results were validated by molecular docking simulation and Western blot (WB) analysis. Finally, we examined the metabolites in rat lung tissues by gas chromatography and mass spectrometry (GC-MS). The results showed that EEAR significantly reduced the W/D ratio, and tumor necrosis factor-α (TNF-α), interleukin-1 beta (IL-1β), interleukin-6 (IL-6) levels in the lungs of ALI model rats. Nineteen components of EEAR were identified and shown to act synergetically by regulating shared pathways such as the mitogen-activated protein kinase (MAPK) and phosphoinositide 3-kinase (PI3K)-protein kinase B (AKT) signaling pathways. Ferulic acid, 4-methylumbelliferone, acetylatractylodinol, atractylenolide I, and atractylenolide III were predicted to bind well to PI3K, AKT and MAPK1, respectively, with binding energies < -5 kcal/mol, although only atractylenolide II bound with high affinity to MAPK1. EEAR significantly inhibited the phosphorylation of PI3K, AKT, p38, and ERK1/2, thus reducing protein expression. EEAR significantly modulated the expression of metabolites such as D-Galactose, D-Glucose, serine and D-Mannose. These metabolites were mainly concentrated in the galactose and amino acid metabolism pathways. In conclusion, EEAR alleviates ALI by inhibiting activation of the PI3K-AKT and MAPK signaling pathways and regulating galactose metabolism, providing a new direction for the development of drugs to treat ALI.

## 1 Introduction

Acute lung injury (ALI) is characterized by pulmonary diffusion dysfunction caused by a variety of direct or indirect factors, such as trauma, infection, noxious gas inhalation, and shock (Nieman et al., 2018). The clinical manifestations are intractable hypoxemia, alveolar and parenchymal edema and progressive respiratory distress (Englert et al., 2019). If the disease is not controlled, it is likely to progress to acute respiratory distress syndrome (ARDS) and multi-organ failure, which is a common critical clinical condition that is a serious threat to human health. Most of the current treatment options, such as dexamethasone, prednisolone, and prednisone reduce inflammation and suppress respiratory failure, but can cause various adverse reactions, including coagulation disorders, gastric ulcers, or osteoporosis (Nieman et al., 2020; Huang et al., 2021). Despite technological advances and the emergence of new treatments, the mortality rate of ALI remains high at 30%–50% (Derwall et al., 2018).

The core theory of the mechanism of ALI is that an imbalance in the inflammatory response exacerbates epithelial or endothelial damage resulting in protein-rich edema fluid entering the alveoli (Fan et al., 2018; Lee et al., 2021). Histologically, ALI is characterized by a severe acute inflammatory response, massive apoptosis of alveolar epithelial cells, a profound increase in alveolar-capillary permeability and subsequent formation of fibrosis (Hakansson et al., 2012). The cytopathology of ALI includes disruption of alveolar-capillary membrane integrity and excessive neutrophil migration as well as increased production and secretion of inflammatory cytokines (Fanelli and Ranieri, 2015).

In recent years, network pharmacology has emerged worldwide as an approach that is based on theories from disciplines such as systems biology, genomics, and proteomics. Network pharmacology uses technologies such as high-throughput genomic data analysis, computer simulation and network database searches to reveal the network relationship of drug-gene-target-disease interactions and predict the mechanism of action of drugs based on the network relationship (Luo et al., 2020). Since Traditional Chinese Medicine (TCM) is characterized as a complex multi-component, multi-target, holistic approach to treating diseases, network pharmacology technology has been adopted to study the material basis of drug efficacy and molecular mechanism of action (Zhang et al., 2019).

In addition, metabolomics has gained popularity as an approach to studying the effects of drugs and diseases based on the use of modern analytical techniques to detect the changes of metabolites in biological systems (Bujak et al., 2015; Wishart, 2019). This approach has also been employed to reveal the mechanism of action of TCMs (Wang et al., 2017).

As a traditional Chinese herb, *Atractylodes lancea* (Thunb.) DC. is commonly used clinically to treat lung diseases and gastrointestinal diseases (Lin et al., 2022; Shi et al., 2022). Atractylodis rhizoma is the main component of the prescription “Mahuang Cangzhu decoction” recorded in “Lan Shi Mi Zang”, which is used to treat cough, sputum, wheezing and dyspnea, symptoms that are similar to those of ALI. Studies have suggested that Atractylodis rhizoma and its extracts have good anti-inflammatory activity and protective effects on epithelial cell barrier integrity (Shi et al., 2019), which would be beneficial to the pathology of ALI. Therefore, in this study, we clarified the efficacy and mechanism of Atractylodis rhizoma in the treatment of a lipopolysaccharide (LPS)-induced ALI model in rats to provide a basis for the development of new therapeutic drugs.

## 2 Materials and methods

### 2.1 Preparation of extract of Atractylodis rhizoma

Samples of Atractylodis rhizoma prepared using a previously described method (Qu et al., 2022) were purchased from Tianji Company (Wuhan, China). The authenticity of samples was confirmed by Professor Xiuqiao Zhang of Hubei University of Traditional Chinese Medicine. Before extraction (three times for 2 h per extraction) in an ultrasonic water bath (25°C), a total of 200 g of *Atractylodis rhizoma* was crushed and soaked overnight in 10-fold volume of 80% ethanol. The extracts were then filtered and concentrated in a rotary evaporator. The final product was weighed under vacuum to calculate the ethanol extract (%) = weight of dried ethanolic extract powder of Atractylodis rhizoma/weight of raw Atractylodis rhizoma herb × 100%

### 2.2 Conversion of dosage

We calculated the experimental doses of the ethanol extract of Atractylodis rhizoma (EEARL) according to the human and rat drug dose relationship of human and rat as previously described (Shi et al., 2022). According to the yield of the ethanol extract of 34%, the low and high doses of EEARL were calculated as 314.7 mg/kg and 2517.4 mg/kg, respectively.

### 2.3 Sample pretreatment and UPLC-MS chromatographic conditions

Sample pretreatment was conducted as previously described (Qu et al., 2022). Briefly, ethanol extract of Atractylodis rhizoma powder (0.2 g) was placed into a conical flask, and 10 times the volume of methanol was added for ultrasonic water bath extraction (25°C). The extract was then centrifuged (5,000 ×*g*) for 10 min at 4°C and the supernatant was filtered (0.22 μm pore size) until further use was required.

The standards atractylodin (PU02410025), chlorogenic acid (PS01311000), ferulic acid (PS01911000), acetylatractylodinol (PS22120806), 4-methylumbelliferone (PS020119), and atractylenolides I (PS010510), II (PS010511), and III (PS010512) were purchased from Chengdu Push Biotechnology Co., Ltd., (Chengdu, China). All standards were dissolved in methanol at a concentration of 1 mg/mL.

UPLC was performed with the Agilent 1,260 Infinity system. We used a Welch Xtimate C18 column (100 mm × 2.1 mm; 1.8 µm) for the separation at 25°C. The mobile phase was acetonitrile (B)-aqueous formic acid (100:0.1, v/v) (A), delivered at a flow rate of 0.3 mL/min, using an injection volume of 5 µL. The elution conditions were set as follows: 20%–50% B: 0–15 min; 50%–60% B: 15–30 min; 60%–95% B: 30–35 min; 95% B: 35–45 min; 95%–20% B: 45–46 min; 20% B: 46–56 min.

We used an LTQ-Orbitrap-MS spectrometer for the mass spectrometry as previously described (Wang et al., 2021). The ion source was electron spray ionization (ESI) and nitrogen was used as the drying gas and atomization gas at flow rates of 10.0 L/min and 3.0 L/min, respectively. Air was used as the heating gas and argon as the collision gas. The desolvent tube temperature was set at 300°C, the heating block temperature at 400°C and the interface temperature at 300°C. The MS scan was operated in scan mode at m/z 100–1,000 and MS 2 at m/z 50–500; 50–1,000.

### 2.4 Animal experiment design

All male Sprague-Dawley (SD) rats (130 ± 10 g) were purchased from the Three Gorges University (Yichang, China). The animal license is No. SCXK (E) 2022-0017. All procedures in the experiments met the ethical requirements and were approved by the Ethics Committee of Hubei University of Traditional Chinese Medicine (approval code: NO.00273381, 9 November 2021). Rats were housed in an environment maintained at 24°C ± 1°C and humidity of 45%–60%.

Rats were treated as previously described (Shi et al., 2022). Briefly, 50 SD rats were randomly divided into five groups: Control group, Model group, EEARL (314.7 mg/kg) group, EEARH (2517.4 mg/kg) group, and Dexamethasone (Dex) (5 mg/kg) group. All drugs were dissolved with normal saline. On days 1–7, normal saline was administered daily by gavage to the Normal and Model groups and the drugs were administered to the other groups by the same route. On day 7, LPS (5 mg/kg) was administered *via* intratracheal instillation to all groups (except the normal group) 2 h after saline or drug administration by gavage (Yang C. et al., 2021). After 6 h, the animals were euthanized with sodium pentobarbital. Samples were then collected until further use was required.

### 2.5 Lung wet/dry ratios

At the end of alveolar lavage, the whole left lung was removed, washed three times with normal saline, blotted with filter paper to remove the surface moisture and immediately weighed to determine the wet weight. The left lung was dried at 80°C for 48 h before the dry weight was recorded. The W/D weight ratio was then calculated.

### 2.6 Detection of inflammatory factors

Bronchoalveolar lavage fluid (BALF) was centrifuged to obtain the supernatant. The expression of TNF-α, IL-1β, IL-6 and monocyte chemoattractant protein-1 (MCP-1) in the supernatant was measured by enzyme-linked immunosorbent assay (ELISA) kits [TNF-α (RK00029), IL-6 (RK00020), and IL-1β (RK00009) purchased from ABclonal Technology Co., Ltd. (Wuhan, China) and MCP-1 (SEKR0024) purchased from Beijing Solarbio Science & Technology Co., Ltd., (Beijing, China)] according to the manufacturers’ instructions.

### 2.7 Immunohistochemical (IHC) staining

Immunohistochemical (IHC) staining of TNF-α, IL-6 and IL-1β expression in lung tissues was performed as previously described (Shi et al., 2022). Briefly, lung tissues were fixed in 4% paraformaldehyde solution for 48 h before incubation with primary antibodies for the detection of TNF-α (ABclonal Technology Co., Ltd., Wuhan, China, A11534) and IL-6 (ABclonal, A21264) and IL-1β (Beijing Solarbio Science & Technology Co., Ltd. (Beijing, China), K108840P). The secondary antibody was purchased from Servicebio Technology Co., Ltd., (Wuhan, China).

### 2.8 Western blot analysis

Western blot analysis was performed as previously described (Shi et al., 2022). Briefly, lung tissue was ground at low temperature and the protein concentration was measured with bicinchoninic acid (BCA) kit (Elabscience Biotechnology Co., Ltd., Wuhan, China). Proteins (15 μg) were separated by sodium dodecyl sulfate-polyacrylamide gen electrophoresis and transferred onto a PVDF membrane. The membrane was blocked with 5% non-fat dried milk in Tris-Buffered Saline with 0.1% Tween 20 (TBST) for 1–2 h at room temperature. After washing three times with TBST, the membranes were incubated with primary antibodies (1:1,000) overnight at 4°C. The membrane was washed three times with TBST and then incubated with secondary antibodies (1:5,000) for 2 h at room temperature. Protein bands were visualized using an enhanced chemiluminescence detection kit (Vazyme Biotech Co., Ltd., Nanjing, China).

Antibodies for the detection of P38 (8690S), p-P38 (4511S), Erk1/2 (4695S), p-Erk1/2 (4370S), AKT (4691S), p-AKT (4060S), PI3K (4249S), β-actin (3700S) and p-PI3K (17366S) were obtained from Cell Signaling Technology, Inc. (Danvers, MA, United States).

### 2.9 Hematoxylin-eosin (HE) staining

Rat lung tissues were fixed in a 4% formaldehyde solution, embedded in paraffin and stained with HE.

### 2.10 Compound screening

The Isomeric SMILES representations of each compound were obtained from PubChem (https://pubchem.ncbi.nlm.nih.gov/) and then entered into SwissADME (http://swissadme.ch/) (Daina et al., 2014; 2017). Screening was performed based on the results of Lipinski, Ghose, Veber, Egan, Muegge and gastrointestinal absorption analyses. Compounds were eliminated if Lipinski, Ghose, Veber, Egan, Muegge showed less than four “Yes” results or gastrointestinal absorption was low.

### 2.11 Target prediction

The targets of the remaining compounds were retrieved through the TCMSP (https://old.tcmsp-e.com/), SwissTargetPrediction (http://www.swisstargetprediction.ch/) (Daina et al., 2019), and DrugBank (https://www.drugbank.com/) databases. All targets were summarized and duplicates were deleted. The targets of ALI were identified by searching the OMIM (https://www.omim.org/), GeneCards (https://www.genecards.org/) and DrugBank (https://www.drugbank.com/) databases. All targets were then summarized and duplicates were deleted.

### 2.12 Network construction

Targets were analyzed in the STRING database (Szklarczyk et al., 2021) (https://cn.string-db.org/, interaction score >0.9 implies high confidence) to obtain the PPI network-related information. Visual analysis was then performed on Cytoscape 3.7.0. Two visualized networks were constructed: (1) Target-Target network (PPI). (2) Compounds-Targets-Pathways network. All networks were constructed by Cytoscape 3.7.0.

### 2.13 Pathway enrichment analysis

To explore the mechanism underlying the effects of EEAR on ALI, GO and KEGG analyses were performed by using the DAVID (https://david.ncifcrf.gov/) (Sherman et al., 2022). Visual analysis was then performed in Omicshare (https://www.omicshare.com/).

### 2.14 Molecular docking simulation of component targets

Molecular docking simulation of component targets was performed as previously described (Eberhardt et al., 2021). Briefly, we obtained 3D structures from the RSCB PDB database (https://ww.rcsb.org/) and used AutoDock Tools version 1.5.6 for molecular docking analysis, which was visualized in PyMol software.

### 2.15 GC-MS analysis of the endogenous metabolites

GC-MS analysis of the endogenous metabolites was performed as previously described (Qu et al., 2022). Briefly, rat lung tissues were placed in EP tubes, ground with methanol and centrifuged. The supernatant was collected and dried under nitrogen, before methoxypyridine solution was added, dried, mixed and centrifuged, and incubated in 80°C water bath for 15 min. Subsequently, bis-trimethylsilyl-trifluoroacetamide (BSTFA) was added, mixed centrifuged, and incubated in 80°C water bath for 15 min. Finally, the solution was centrifuged and supernatant was collected for GC-MS analysis using a DB-5MS capillary column (30.0 µm × 250 µm, 0.25 µm) under the following conditions: initial temperature of 80°C for 3 min, ramped to 140°C at a rate of 7°C/min for 4 min, then to 180°C at a rate of 5°C/min for 6 min, then to 280°C at a rate of 5°C/min for 2 min. Helium was used as carrier gas delivered at a flow rate of 1 mL/min and a shunt ratio of 10:1. The injector temperature was 280°C. The ion source temperature was 200°C. The MS scan was operated at 280°C in scan mode at m/z 50–650. The data matrix was analyzed using Metaboanalyst 5.0 (https://www.metaboanalyst.ca/) and biomarkers were screened by VIP >1 and *p* < 0.05.

### 2.16 Statistical analysis

Data were expressed as means ± standard error of the mean (SEM) and analyzed by a one-way ANOVA, followed by Dunnett’s *post hoc* test. Statistical analysis was performed using GraphPad 8.0. *p* < 0.05 was set as the threshold for statistical significance.

## 3 Results

### 3.1 Effect of EEAR on the W/D ratio and pathological changes in the lungs of ALI model rats

**FIGURE 1:**
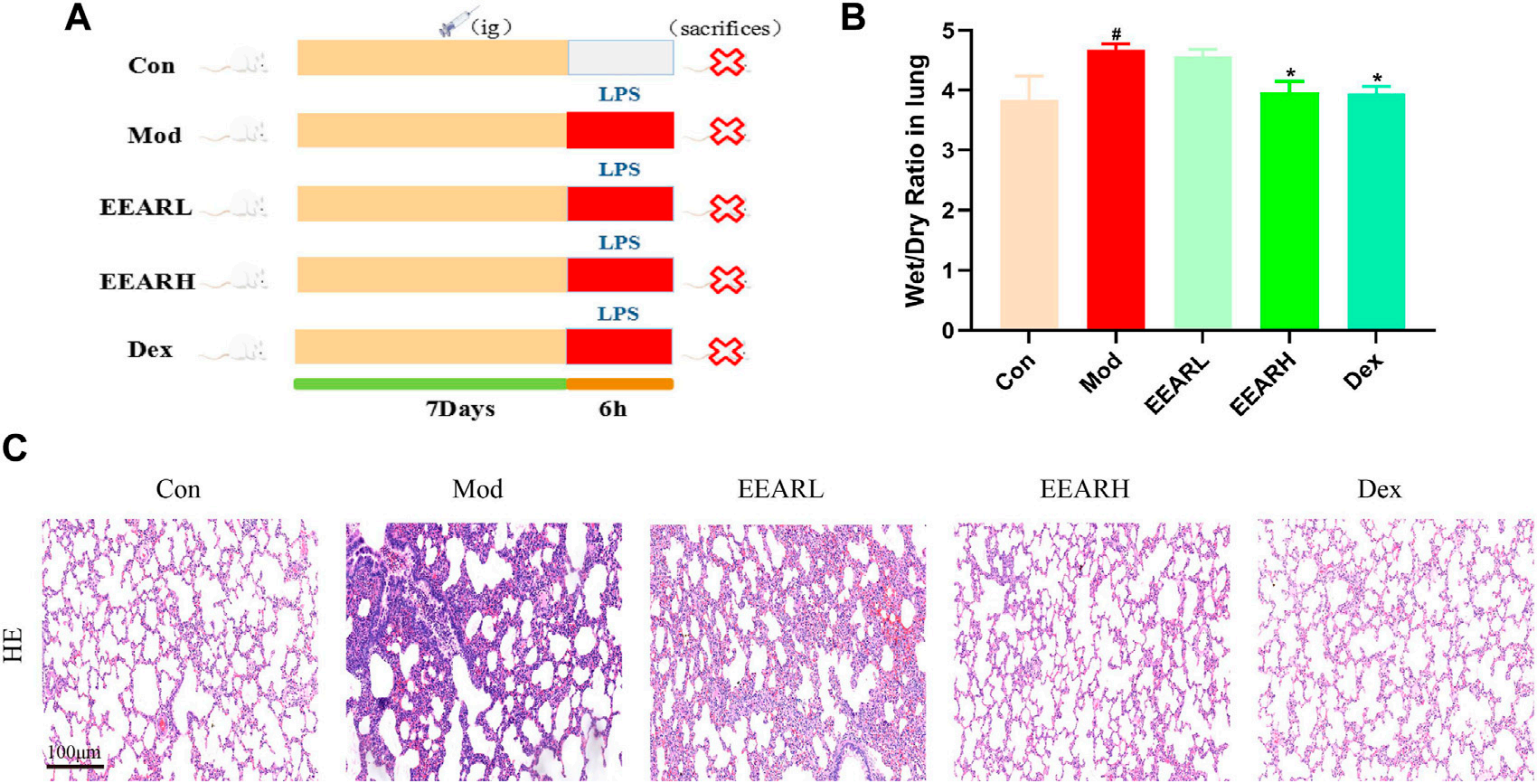
EEAR attenuated pulmonary pathological changes and reduced the W/D ratio. **(A)** Schematic diagram of the experimental process. **(B)** Lung W/D ratio (n = 10). **(C)** Hematoxylin-eosin (HE) staining. Data represent means ± SEM. ^*#*^ *p* < 0.05 vs. Control; ^*^ *p* < 0.05 vs. Model.

To evaluate the effect of EEAR in alleviating ALI, we conducted the experiment shown in Figure 1A. The results showed that the W/D ratio in the Model group increased significantly compared with the Control group (*p* < 0.05), while there was a significant decrease in the EEARH group (*p* < 0.05) (Figure 1B). Evaluation of the HE stained sections revealed significant alveolar wall thickening and inflammatory cell aggregation in the Model group, while improvements in the pathological changes were observed in the EEARH group, with decreased inflammatory cell aggregation, and reduced alveolar wall thickening. EEARL also decreased the aggregation of inflammatory cells, although the effect was not as great as that observed in the EEARH group (Figure 1C). Thus, these findings demonstrated that EEAR attenuated pulmonary pathological changes and reduced the W/D ratio in ALI rats.

### 3.2 Effect of EEAR on pulmonary inflammation in rats with ALI

**FIGURE 2:**
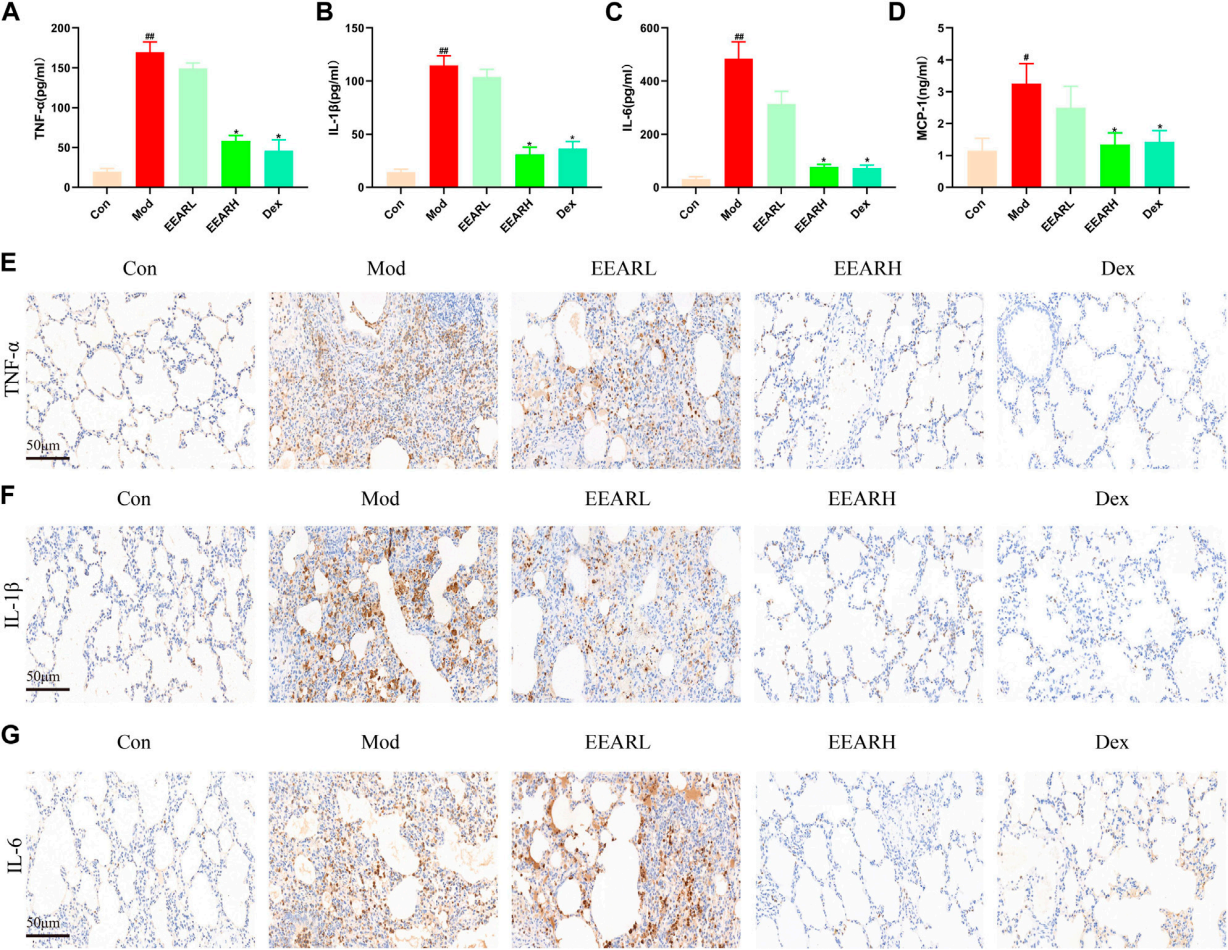
EEAR reduces lung inflammation in rats. **(A)** TNF-α expression in the BALF. **(B)** IL-1β expression in the BALF. **(C)** IL-6 expression in the BALF. **(D)** MCP-1 expression in the BALF. **(E)** Immunohistochemical staining of TNF-α. **(F)** Immunohistochemical staining of IL-1β. **(G)** Immunohistochemical staining of IL-6. Data are expressed as means ± SEM, n = 10. ^*##*^ *p* < 0.01 vs. Control; ^*#*^ *p* < 0.05 vs. Control; ^*^ *p* < 0.05 vs. Model.

Since inflammation is closely related to ALI (Shen et al., 2017), we also examined the level of lung inflammation in rats with ALI. The expression levels of inflammatory factors were significantly higher in the Model group compared with those in the Control group (*p* < 0.01) (Figures 2A–D), while the expression levels in the EEARH group were significantly reduced (*p* < 0.05). The expression levels of these inflammatory factors were also reduced in the EEARL group, although the effects were not statistically significant. In addition, IHC analysis showed that the expression of TNF-α, IL-1β, and IL-6 were significantly increased in the Model group, while the expression of all these factors was decreased to varying degrees after drug administration, with the most significant effects observed in the EEARH and Dex groups (Figures 2E–G).

### 3.3 Chemical composition of EEAR

**FIGURE 3:**
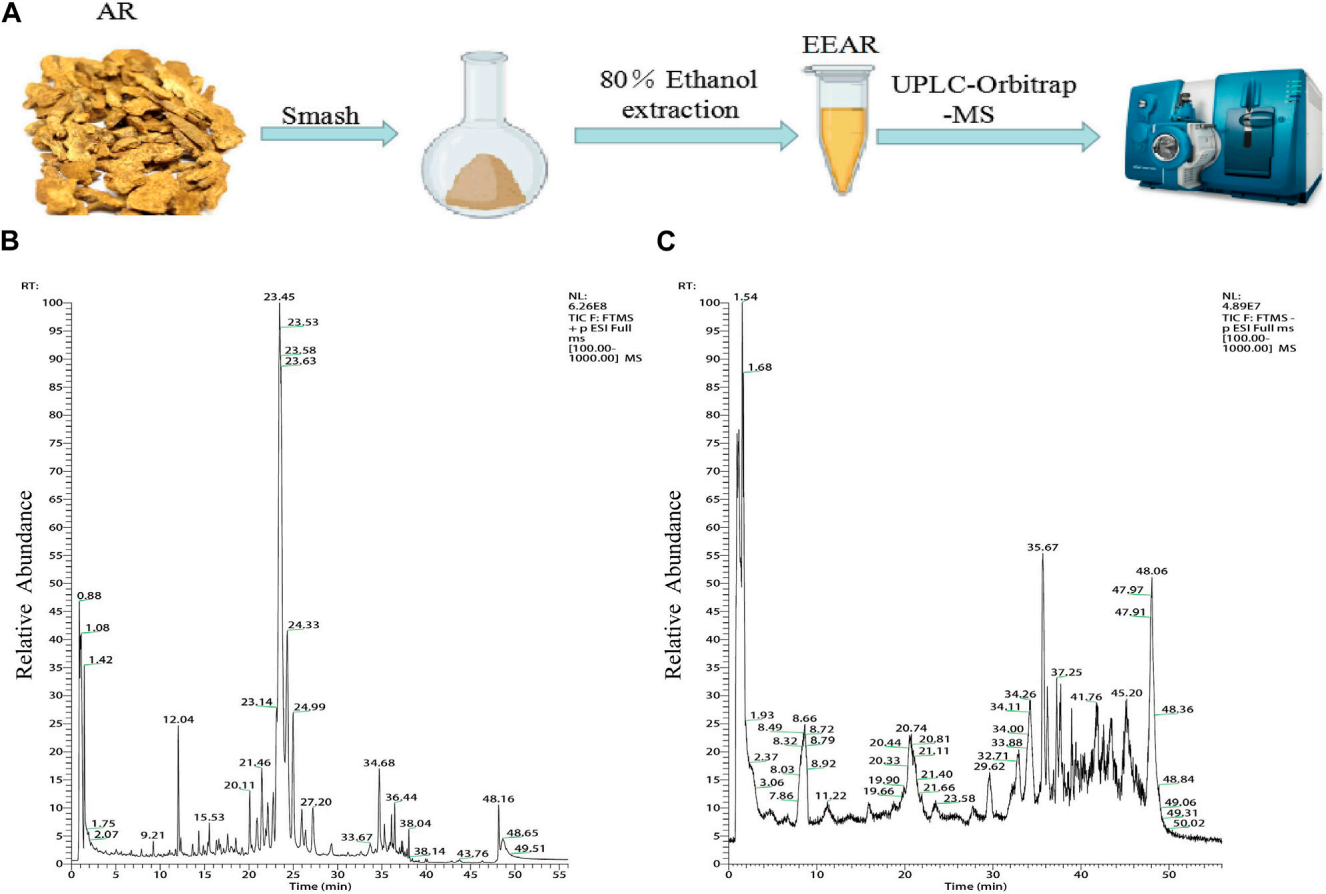
Chemical composition analysis of EEAR determined by UPLC-MS. **(A)** Schematic diagram of the experimental process. **(B)** Chromatogram in positive ion mode. **(C)** Chromatogram in negative ion mode.

We characterized the composition of EEAR by UPLC-MS (Figures 3A–C). A total of 19 compounds were identified by comparison with standard product information, published reports and mass spectrometry libraries (Table 1 and Supplementary Table S1).

**TABLE 1:** 19 compounds identified by UPLC-MS.

NO.	Compound	Formula
C1	Chlorogenic acid^*^	C_16_H_18_O_9_
C2	Ferulic acid^*^	C_10_H_10_O_4_
C3	β-vetivenene	C_15_H_22_
C4	β-farnesene	C_15_H_24_
C5	Atractylenolide III^*^	C_15_H_20_O_3_
C6	Atractylodin^*^	C_13_H_10_O
C7	Atractylenolactam	C_15_H_19_NO
C8	Diacetyl-atractylodiol	C_17_H_20_O_4_
C9	Aractylenolide II^*^	C_15_H_20_O_2_
C10	4-Methylumbelliferone^*^	C_10_H_8_O_3_
C11	Acetylatractylodinol^*^	C_15_H_12_O_3_
C12	Beta-Caryophyllene	C_15_H_24_
C13	Atractylenolide I^*^	C_15_H_18_O_2_
C14	Alpha-Guaiene	C_15_H_24_
C15	Selina-4(15),7(11)-Dien-8-One	C_15_H_22_O
C16	β-eudesmol	C_15_H_26_O
C17	3β-acetoxyatractylone	C_17_H_22_O_3_
C18	2-phenyl-anisol	C_13_H_12_O
C19	Atractylone	C_15_H_20_O

Note: The substances with * in the table have been compared with the reference substance.

### 3.4 Network pharmacology analysis

**FIGURE 4:**
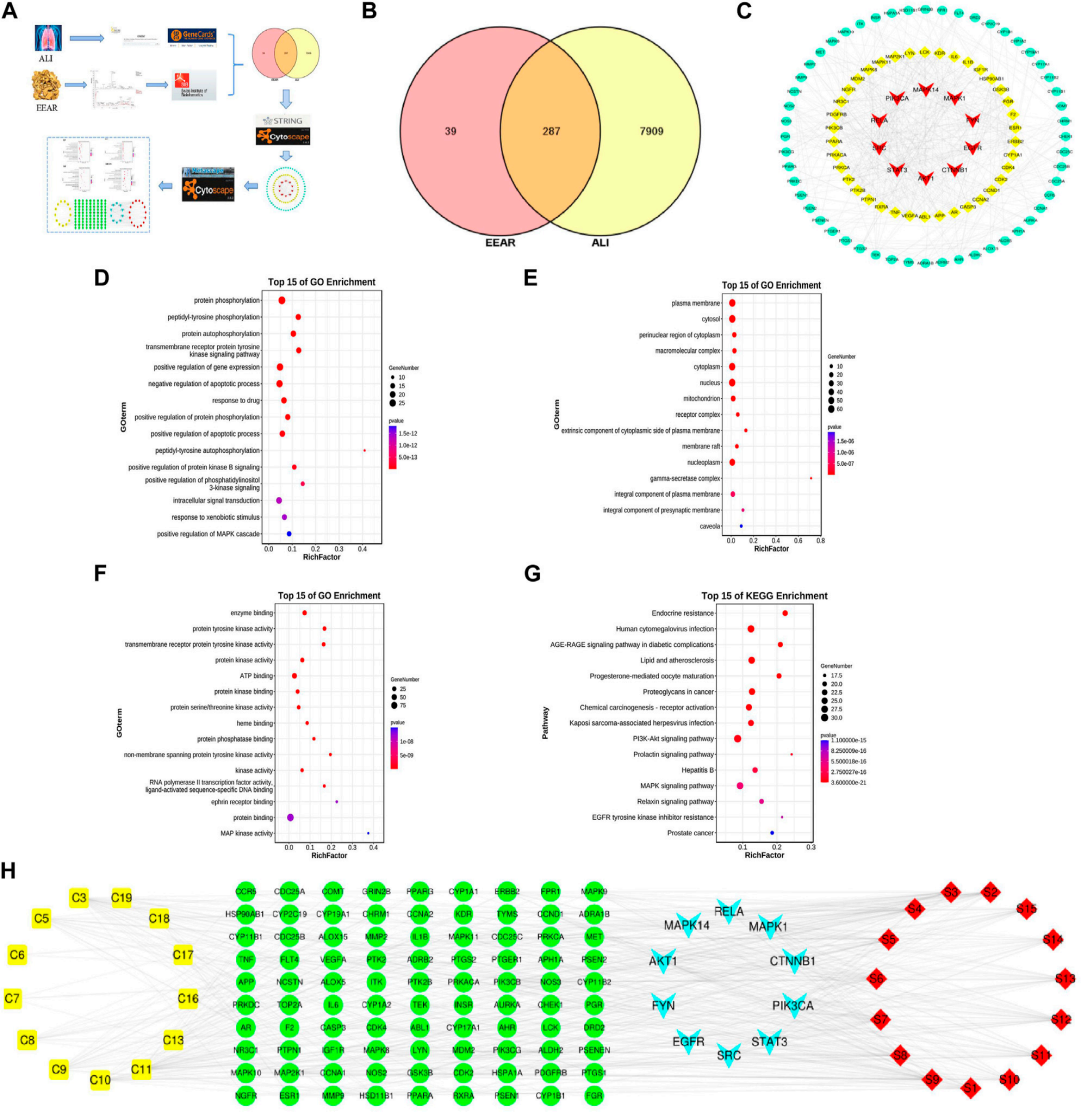
Network pharmacology analysis of EEAR. **(A)** Schematic diagram of the experimental process. **(B)** Venn diagram of disease targets and compounds targets. **(C)** PPI association chart of the top 100 targets. **(D)** Biological processes analysis of the top 100 targets. **(E)** Cellular components analysis of the top 100 targets. **(F)** Molecular functions analysis of the top 100 targets. **(G)** KEGG analysis of the top 100 targets. **(H)** Compounds-targets-pathways network map. Compounds are shown in yellow; targets in green and light blue and the signaling pathways in red.

To clarify the mechanism by which EEAR alleviates ALI, we used network pharmacology for further analysis of the UPLC-MS data. First, we entered the data for each compound into the SwissADME database and screened the compounds based on the results of Lipinski, Ghose, Veber, Egan, Muegge and gastrointestinal absorption analyses. Based on the screening criteria, we identified 13 eligible compounds (Supplementary Table S2). We then retrieved the targets of these 13 compounds by screening the TCMSP, SwissTargetPrediction and DrugBank databases, and finally obtained 326 targets. By screening the GeneCards, DrugBank, and OMIM databases, we retrieved a total of 8,196 targets associated with ALI. By intersecting disease targets with compound targets, we obtained a total of 287 targets (Figure 4B), which were uploaded to the STRING database for analysis, and then visualized in Cytoscape. The top 100 targets were selected according to the degree-value ranking obtained in the association analysis, and the core targets included SRC, STAT3, PIK3CA, and MAPK1 (Figure 4C; Supplementary Table S3).

To investigate the biological functions of the target genes, we performed three GO functional analyses on the top 100 targets. This approach identifies targets enriched in the categories of biological processes, cellular components and molecular functions. The targets were mainly enriched in protein phosphorylation, signaling and regulation of apoptotic processes, protein kinase binding and protein kinase activity (Figures 4D–F). The results of KEGG analysis indicated that these targets were enriched in pathways such as lipids and atherosclerosis, and the PI3K-Akt and MAPK signaling pathways (Figure 4G; Supplementary Table S4).

To further clarify the mechanism underlying the therapeutic effects of EEAR in ALI, we constructed “compounds-targets-pathways” network map using Cytoscape. We filtered the results by degree-value ranking and found that the top ranked compounds were ferulic acid, acetylatractylodinol, 4-methylumbelliferone, and atractylenolides I, II, and III. The top ranked targets, MAPK1, AKT1, PIK3CA and MAPK14, were implicated as the key targets of EEAR for the treatment of ALI. The top ranked signaling pathways included PI3K-AKT the MAPK signaling pathways (Figure 4H).

### 3.5 Molecular docking simulation and WB validation

**FIGURE 5:**
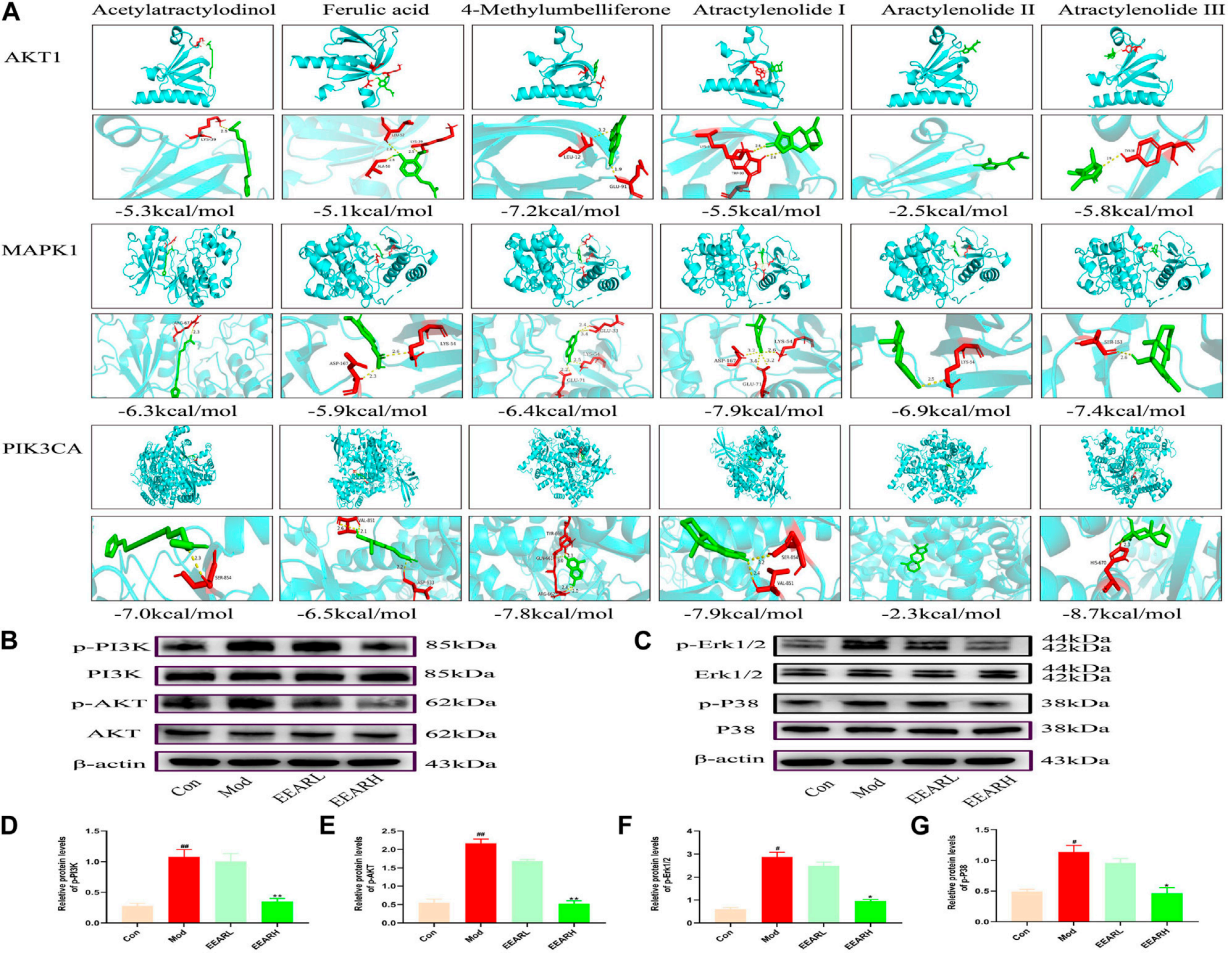
Molecular docking simulation and WB verification. **(A)** Molecular docking simulation of compounds and targets. **(B)** Western blot analysis of PI3K-AKT signaling pathway expression levels in lung tissue. **(C)** Western blot analysis of MAPK signaling pathway expression levels in lung tissue. **(D)** Quantitative analysis of the p-PI3K levels. **(E)** Quantitative analysis of p-AKT levels. **(F)** Quantitative analysis of p-Erk1/2 levels. **(G)** Quantitative analysis of p-P38 levels. Data represent means ± SEM (n = 3). ^*##*^ *p* < 0.01 vs. Control; ^*#*^ *p* < 0.05 vs. Control; ^**^ *p* < 0.01 vs. Model; ^*^ *p* < 0.05 vs. Model.

We next further validated the results of the network pharmacology analysis, by performing molecular docking simulation. This technique predicts the potential for binding between the target and compound, and assesses the affinity between the compound and the target protein by comparing the docking fraction with that of the original ligand. We selected the top six compounds and the top three targets for molecular docking simulation. All six compounds were predicted to bind to with high affinity to MAPK1 (PDB ID:6RQ4), with binding energies < −5 kcal/mol. With the exception of atractylenolide II, the other five compounds bound with high affinity to AKT1 (PDB ID:6HHF) and PIK3CA (PDB ID:7K6N), with binding energies < −5 kcal/mol. Therefore, we identified MAPK1, AKT1, and PIK3CA as the main potential targets of EEAR in the treatment of ALI (Figure 5A).

We further verified the results of molecular docking simulation by WB analysis. The expression levels of phosphorylated PI3K, AKT, Erk1/2 and P38 were significantly increased in the Model group (*p* < 0.01) and decreased to varying degrees after EEAR treatment, with the most significant decrease in the EEARH group (*p* < 0.01) (Figures 5B–G). Therefore, combined with the previous results, we proposed that EEAR alleviates ALI by inhibiting the activation of the PI3K-AKT and MAPK signaling pathways.

### 3.6 Metabolomic analysis

#### 3.6.1 Multivariate statistical analysis

**FIGURE 6:**
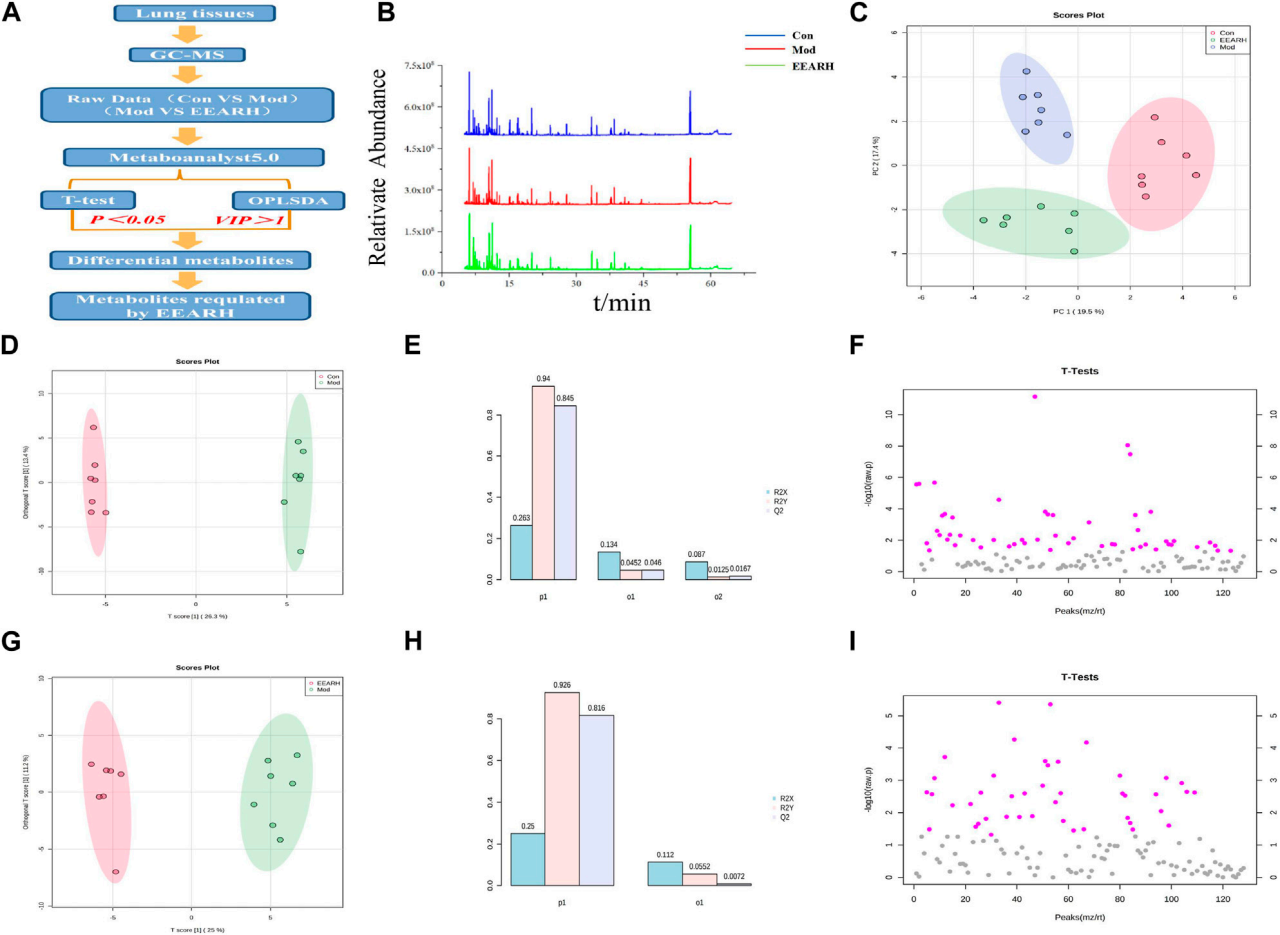
Multidimensional analysis of metabolite data. **(A)** Schematic diagram of the experimental process. **(B)** Metabolic profiles of rat lung tissue samples. **(C)** Metabolite PCA scores. **(D)** OPLS-DA analysis of metabolites in the Control and Model groups. **(E)** OPLS-DA model parameters for the Control and Model groups. **(F)** Student’s t*-*test analysis of metabolites in the Control and Model groups. **(G)** OPLS-DA analysis of metabolites in the EEARH and Model groups. **(H)** OPLS-DA model parameters for the EEARH and Model groups. **(I)** Student’s t-test analysis of metabolites in the EEARH and Model groups (n = 7).

We next investigated the effect of EEAR on the metabolism of LPS-induced ALI rats using a metabolomics approach to detect changes in metabolites in the lung tissue. High-dose EEAR appeared to have the greatest effect on ALI; therefore, this dose was selected for use in our subsequent research. A schematic diagram of the metabolomics analysis process is shown in Figures 6A,B. Principal component analysis (PCA) revealed a clear separation between the Control, Model, and EEARH groups, indicating differences in the metabolites in these groups. However, the distance between the Control and EEARH groups was less marked than that between the Control and Model groups, indicating a higher degree of similarity between the levels and types of metabolites in these groups (Figure 6C). To further elucidate the ability of EEAR to regulate metabolites, the differential metabolites and metabolic pathways associated with ALI disease were identified based on VIP >1 in the orthogonal projection to latent structures square discriminate analysis (OPLS-DA) model combined with *p* < 0.05 in Student’s t-test (Figures 6F,I). The established OPLS-DA model showed good discrimination between the Control and Model groups. Similarly, the OPLS-DA model showed good discrimination between the EEARH and Model groups (Figures 6D,G). In addition, the good predictive performance of the OPLS-DA model developed in this study was confirmed in 100 external experiments (Figures 6E,H).

#### 3.6.2 Metabolic pathways analysis and targets analysis

**FIGURE 7:**
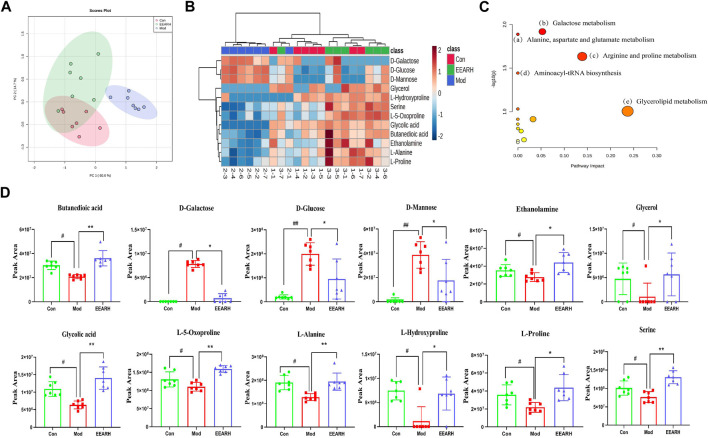
Metabolic pathways and targets analysis. **(A)** PCA of 12 differential metabolites. **(B)** Heat map analysis. **(C)** Enrichment analysis of metabolites. **(D)** Statistical analysis of 12 differential metabolites. Data represent means ± SEM, n = 7. ^*##*^ *p* < 0.01 vs. Control; ^*#*^ *p* < 0.05 vs. Control; ^**^ *p* < 0.01 vs. Model; ^*^ *p* < 0.05 vs. Model.

Based on VIP >1 in the OPLS-DA model combined with *p* < 0.05 in Student’s t-test, we obtained a total of 53 differential metabolites by comparison of the Control and Model groups. Similarly, we obtained a total of 22 differential metabolites by comparison of the Model and EEARH groups using the same criteria. By intersection of the obtained metabolites, we identified 18 metabolites that were commonly present (Supplementary Table S5), 12 of which were found to be present at significantly different levels after treatment. PCA showed that the Model group clustered into a single category, while there was a partial overlap between the Control and EEARH groups, indicating a higher degree of similarity between the metabolite levels in these two groups (Figure 7A). Clustering heat map analysis also showed almost complete clustering of the Control and EEARH groups into a single group, further confirming the high degree of similarity between the metabolite levels in these two groups (Figure 7B). Metaboanalyst enrichment analysis revealed that these 12 differentially expressed metabolites mainly affected the pathways of galactose metabolism, amino acid metabolism, and aminoacyl-tRNA biosynthesis (Figure 7C). Among these metabolic pathways, galactose metabolism was the most significant (Supplementary Table S6). As shown in Figure 7D, the levels of D-Glucose, D-Mannose and D-Galactose were significantly increased in the Model group compared to those in the Control group (*p* < 0.01), while the levels of Serine, L-Alanine, and L-Proline were significantly decreased (*p* < 0.05). The levels of all of these metabolites recovered to varying degrees after treatment with EEAR.

## 4 Discussion

The inflammatory response is an important pathological mechanism responsible for ALI (Lee et al., 2021). Following lung injury, inflammatory cells are recruited to the site and secrete large amounts of inflammatory factors, which activate effector cells. In ALI, the inflammatory response mechanism is dysregulated and the imbalance of pro- and anti-inflammatory mediators in the lung leads to the exacerbation of the injury (Chen et al., 2020; Ge et al., 2020).

An increasing number of studies have shown that inhibiting the release of pro-inflammatory factors and modulating the immune system can promote the improvement of endothelial function in patients with ALI (Zhang et al., 2016; Lv et al., 2017). Thus, inhibiting the release of pro-inflammatory factors and regulating the immune system to promote the improvement of endothelial function in ALI patients may reduce the mortality rate and improve the prognosis (Walkey and Wiener, 2012).

TCM has great potential to prevent and treat ALI by interfering with the signaling pathways and targets of ALI through different pharmacological effects (Ding et al., 2020). Thus, TCM provides a new therapeutic perspective for the treatment of ALI. As a TCM, *Atractylodis rhizoma* has good anti-inflammatory activity. In the present study, we clarified the therapeutic effect of *A. rhizoma* on ALI by measuring the W/D ratio of the lung, analyzing the expression of inflammatory factors in the lung, and evaluating the pathology in histologically processed tissue sections.

Network pharmacology considers the occurrence and development of diseases as a long-term and complex process caused imbalanced expression of multiple target genes and their products. This approach can be used for systematic analysis of “diseases-targets-drugs” interaction networks and reveal the mechanism of drug synergy. The emergence of network pharmacology has promoted a more accurate understanding of the mode of action and the main active ingredients of TCMs from the holistic “multi-component-multi-target-complex disease” perspective. In this study, we used UPLC-MS to analyze the chemical composition of EEAR and further clarified the mechanism by which EEAR alleviates ALI by network pharmacology analysis. We found that the main active components involved in the mechanism by which EEAR alleviates ALI were identified as ferulic acid, acetylatractylodinol, 4-methylumbelliferone, and atractylenolides I, II and III. In addition, our research indicated that the therapeutic effects of EEAR on ALI may be mediated by regulating the PI3K-AKT and MAPK signaling pathways. Studies have shown that the PI3K-AKT and MAPK signaling pathways are the major intracellular signaling pathways that regulate inflammation and are closely related to LPS-induced ALI (Li et al., 2018; Meng et al., 2018; Nie et al., 2019). Furthermore, inhibition of upstream protein expression in these pathways effectively reduces the release of inflammatory factors and attenuates the inflammatory response. The results in molecular docking simulation and WB verification were consistent with these findings. We found that EEAR effectively inhibited the phosphorylation of PI3K, AKT, P38, and Erk1/2. Therefore, we hypothesize that EEAR alleviates ALI by inhibiting activation of the MAPK and PI3K-AKT signaling pathways.

Metabolomics techniques have been used to study metabolites associated with ALI in animals and patients with the aim of identifying disease-related biomarkers and novel drug targets (Ding et al., 2020). For example, Huanglian Jiedu Decoction was shown to alleviate ALI by regulating the sphingolipid pathway to inhibit NLRP3 inflammasome activation (Chen et al., 2022). The metabolomic results obtained in the present study revealed significant changes in 12 metabolites, mainly some sugar and amino acid metabolites, following EEAR treatment of ALI. In the Model group, the levels of D-Glucose, D-Mannose and D-Galactose were significantly increased, while those of amino acids such as Serine, L-Alanine and L-Proline were significantly decreased. The levels of all of these metabolites were changed after the administration of EEAR. Since amino acids function as regulators of inflammatory processes, disturbances in the metabolism of these amino acids may have implications for inflammation-related diseases (Ji et al., 2022; Piestansky et al., 2022). Thus, we propose that EEAR may alleviate ALI by modulating disturbances in the amino acid metabolic profile.

In addition, we found that EEAR significantly reduced the levels of D-Galactose. Under normal conditions, galactose is converted to glucose, but in excess, it is metabolized to generate ROS and advanced glycation end-products, which act on advanced glycosylation end-product receptors (Abd El-Fatah et al., 2021). These metabolites induce oxidative stress, mitochondrial dysfunction, cellular damage and inflammatory responses (Sun et al., 2018; Yang X. et al., 2021). Previous studies have shown that galactose is associated with oxidative stress, immune inflammation, and metabolic disorders, and can activate inflammatory signaling pathways such as the MAPK, PI3K-Akt, and NF-κB pathways to promote the release of inflammatory factors (Zhong et al., 2020; Chen et al., 2021). Therefore, combined with our previous results, we hypothesize that EEAR mitigates ALI by regulating galactose metabolism to inhibit PI3K-Akt and MAPK signaling pathway activation.

There are few reports on the treatment of ALI by EEAR. In our study, we combined metabolomics and network pharmacology to clarify the effect of EEAR on ALI, and conducted a preliminary study of the mechanism. In addition, we initially clarified the main active components of EEAR for the treatment of ALI. Thus, further research is needed to fully elucidate the mechanism by which EEAR alleviates ALI, and the identity of the component that plays the main role in this process.

## 5 Conclusion

**FIGURE 8:**
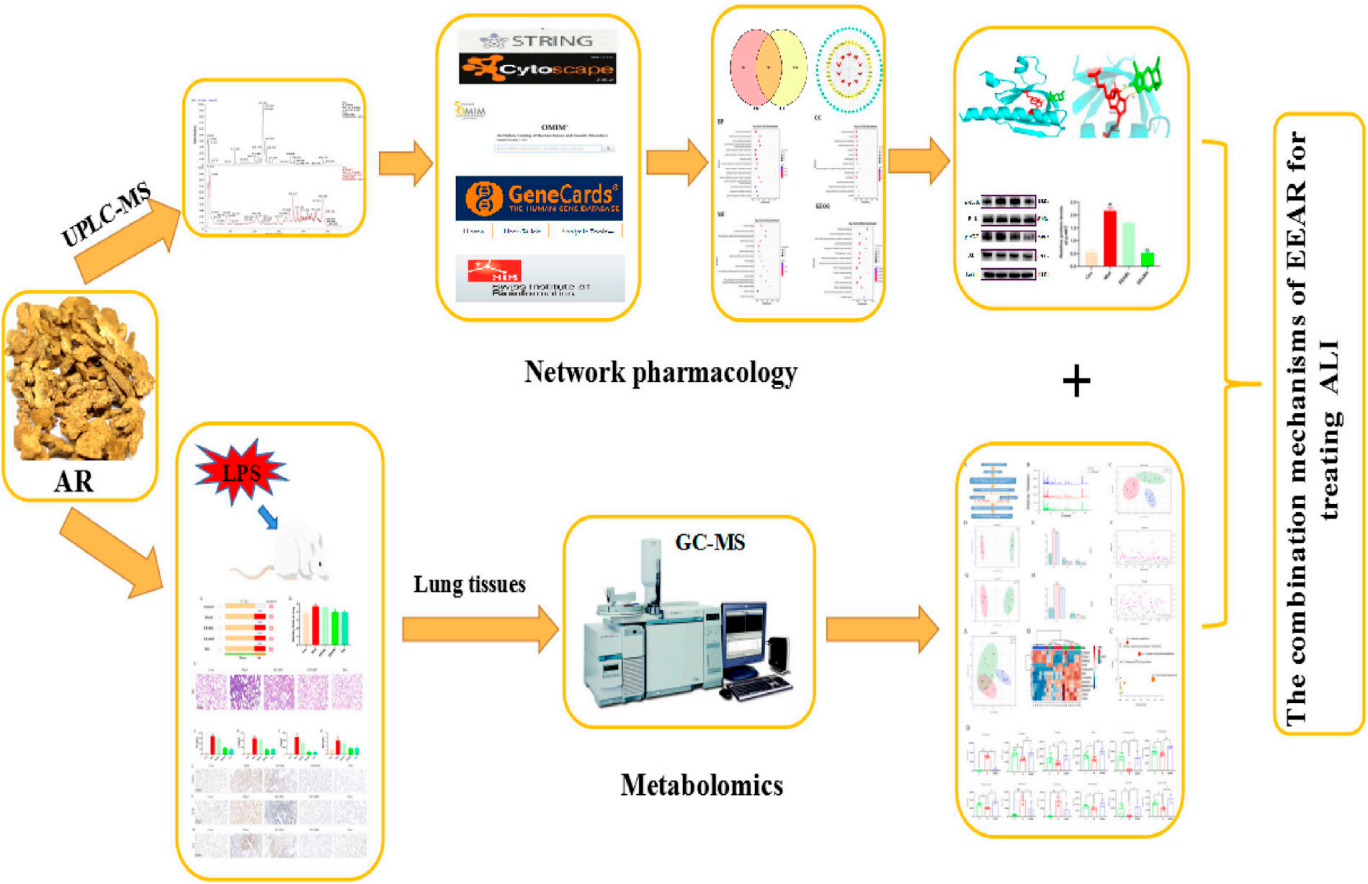
Workflow diagram for elucidation of the mechanisms underlying the therapeutic effects of EEAR in ALI.

In conclusion, the multi-compound, multi-target and multi-mechanism mechanism underlying the therapeutic effects of EEAR on ALI were systematically elucidated by integrating network pharmacology and metabolomics (Figure 8). We provide evidence that EEAR alleviates ALI by regulating galactose metabolism to inhibit activation of the PI3K-AKT and MAPK signaling pathways. These findings further clarify the mechanism underlying the therapeutic effects of *A. rhizoma* in ALI and also provide a rational basis for the clinical application of TCMs.

## Data Availability

The raw data supporting the conclusion of this article will be made available by the authors, without undue reservation.

## Abbreviations

ALI, Acute lung injury; EEAR, ethanolic extract of Atractylodis rhizoma; AR, Atractylodis rhizoma; LPS, lipopolysaccharide; WB, Western blot; HE, hematoxylin and eosin; BCA, bicinchoninic acid; TNF-α; tumor necrosis factor alpha; IL-6, interleukin-6; IL-1β, interleukin-1β; PI3K, phosphoinositide 3-kinase; MAPK, mitogen-activated protein kinases; ARDS, acute respiratory distress syndrome; UPLC, Ultra Performance Liquid Chromatography; ELISA, enzyme-linked immunosorbent assay; TCM, traditional Chinese medicine; IHC, immunohistochemistry; PCA, principal component analysis; MCP-1, monocyte chemotactic protein-1; OPLS-DA, orthogonal projection to latent structures square discriminate analysis.
