# Supplementary material for: Therapeutic effects and mechanism of Atractylodis rhizoma in acute lung injury: Investigation based on an Integrated approach
Source: Front Pharmacol. 2023 Apr 24;14:1181951. doi: 10.3389/fphar.2023.1181951 (PMC10164760; doi:10.3389/fphar.2023.1181951)
Supplement: Supplementary file 1 [file Table1.DOC]

Table S1 Compounds and fragment ion information

| No. | t_R_（min） | Compound | Formula | m/z | Selective ion | Fragment ion（m/z） |
| --- | --- | --- | --- | --- | --- | --- |
| C1 | 1.63 | Chlorogenic acid | C_16_H_18_O_9_ | 353.0878 | [M-H]^-^ | 309.094,295.0803,191.057,179.0358,161.025,143.0419,135.0462,127.039,111.0423,99.0481,5.0339,59.0156 |
| C2 | 3.8 | Ferulic acid | C_10_H_10_O_4_ | 193.0507 | [M-H]^-^ | 178.028,149.0582,134.0365,91.0162,59.0043 |
| C3 | 11.73 | β-vetivenene | C_15_H_22_ | 203.1794 | [M+H]^+^ | 161.1334,147.1170,133.1051,119.0866,95.0865 |
| C4 | 12.32 | β-Farnesene | C_15_H_24_ | 205.1951 | [M+H]^+^ | 149.1325,135.1168,123.1168,121.1013,109.1018,107.0863,95.085,93.0710,81.0714,67.0566,57.0737,55.0580,53.0424 |
| C5 | 15.39 | Atractylenolide III | C_15_H_20_O_3_ | 249.1485 | [M+H]^+^ | 232.14587,231.1374,213.1250,207.13785,203.1423,189.1258,175.0747,163.0746,149.0592,123.0795,119.0861,105.0690,91.0558 |
| C6 | 15.53 | Atractylodin | C_13_H_10_O | 183.0804 | [M+H]^+^ | 168.0578,165.0666,155.0507,153.0679,141.0682,129.0709,127.0530,115.0527,77.037 |
| C7 | 16.36 | Atractylenolactam | C_15_H_19_NO | 230.15394 | [M+H]^+^ | 215.1325,202.1239,185.1308,174.0916,160.0765,143.0844,124.0752,105.0696 |
| C8 | 17.52 | Diacetyl-atractylodiol | C_17_H_20_O_4_ | 289.1434 | [M+H]^+^ | 155.0850,141.0691,117.0704,111.0484,105.0701,271.1446,245.1243,229.1164,183.1224 |
| C9 | 19.25 | Aractylenolide II | C_15_H_20_O_2_ | 233.1536 | [M+H]^+^ | 215.1412,205.1552,197.1298,187.1489,177.0906,159.0802,145.1012,151.0743,131.0857,105.0689,91.0559 |
| C10 | 20.11 | 4-Methylumbelliferone | C_10_H_8_O_3_ | 177.0546 | [M+H]^+^ | 159.0798,149.0550,145.0289,134.0332,121.0985,107.0797,89.0352,77.0381 |
| C11 | 20.87 | Acetylatractylodinol | C_15_H_12_O_3_ | 241.0859 | [M+H]^+^ | 223.07549,213.0864,199.0708,181.0771,171.08057,166.0742,143.0827,129.0702,115.0501,93.0382 |
| C12 | 21.46 | Beta-Caryophyllene | C_15_H_24_ | 205.1956 | [M+H]^+^ | 189.1643,165.1643,163.1487,149.1330,135.1174,123.117 |
| C13 | 22.11 | Atractylenolide I | C_15_H_18_O_2_ | 231.1380 | [M+H]^+^ | 213.1270,203.1085,189.09128,185.1321,175.1091,163.07555,161.05995,157.1006,149.05988,143.0850,129.0692,105.0698,91.0554,81.0700 |
| C14 | 22.77 | Alpha-Guaiene | C_15_H_24_ | 203.1805 | [M-H]^-^ | 187.1492,185.1336,171.1179,159.1179,147.1179 |
| C15 | 23.45 | Selina-4(15),7(11)-Dien-8-One | C_15_H_22_O | 219.1743 | [M+H]^+^ | 201.1628,191.1773,175.1474,159.1162,145.1007,131.0857,123.0803,105.0709,95.085 |
| C16 | 23.52 | β-eudesmol | C_15_H_26_O | 240.2323 | [M+NH_4_]^+^ | 205.1948,183.1736,149.1308,135.1168,97.0986,71.0901,67.0632 |
| C17 | 24.33 | 3β-acetoxyatractylone | C_17_H_22_O_3_ | 275.1647 | [M+H]^+^ | 215.1435,197.1329,145.1016,119.0852 |
| C18 | 25.96 | 2-phenyl-anisol | C_13_H_12_O | 185.0961 | [M+H]^+^ | 170.0725,157.0645,142.0776,129.0695 |
| C19 | 33.67 | Atractylone | C_15_H_20_O | 217.1587 | [M+H]^+^ | 199.1459,189.1244,175.14772,171.1144,161.0944,143.0847,133.10081,123.0794 |

Table S2 Compounds were screened according to the drug-likeness five principles (Lipinski, Ghose, Veber, Egan, Muegge) and gastrointestinal absorption (GI)

| No.CompoundLipinskiGhoseVeberEganMueggeGI |  |  |  |  |  |  |  |
| --- | --- | --- | --- | --- | --- | --- | --- |
| C1 | Chlorogenic acid | YesNoNoNoNolow |  |  |  |  |  |
| C2 | Ferulic acid | YesYesYesYesNolow |  |  |  |  |  |
| C3 | β-vetivenene | YesYesYesYesNohigh |  |  |  |  |  |
| C4 | β-farnesene | YesYesYesYesNolow |  |  |  |  |  |
| C5 | Atractylenolide III | YesYesYesYesYeshigh |  |  |  |  |  |
| C6 | Atractylodin | YesYesYesYesNohigh |  |  |  |  |  |
| C7 | Atractylenolactam | YesYesYesYesYeshigh |  |  |  |  |  |
| C8 | Diacetyl-atractylodiol | YesYesYesYesYeshigh |  |  |  |  |  |
| C9 | Aractylenolide II | YesYesYesYesYeshigh |  |  |  |  |  |
| C10 | 4-Methylumbelliferone | YesYesYesYesNohigh |  |  |  |  |  |
| C11 | Acetylatractylodinol | YesYesYesYesYeshigh |  |  |  |  |  |
| C12 | Beta-Caryophyllene | YesYesYesYesNolow |  |  |  |  |  |
| C13 | Atractylenolide I | YesYesYesYesYeshigh |  |  |  |  |  |
| C14 | Alpha-Guaiene | YesYesYesYesNolow |  |  |  |  |  |
| C15 | Selina-4(15),7(11)-Dien-8-One | NoYesYesYesNohigh |  |  |  |  |  |
| C16 | β-eudesmol | YesYesYesYesNohigh |  |  |  |  |  |
| C17 | 3β-acetoxyatractylone | YesYesYesYesYeshigh |  |  |  |  |  |
| C18 | 2-phenyl-anisol | YesYesYesYesNohigh |  |  |  |  |  |
| C19 | Atractylone | YesYesYesYesNohigh |  |  |  |  |  |

Table S3 The core targets of top 20 and the topological parameters

| No. | Target | Degree | Betweenness centrality | Closeness centrality |
| --- | --- | --- | --- | --- |
| 1 | SRC | 49 | 0.15823205 | 0.42348008 |
| 2 | STAT3 | 34 | 0.10775577 | 0.39920949 |
| 3 | PIK3CA | 33 | 0.04469669 | 0.37757009 |
| 4 | CTNNB1 | 32 | 0.11495274 | 0.39376218 |
| 5 | MAPK1 | 31 | 0.07214663 | 0.40000000 |
| 6 | RELA | 29 | 0.09159469 | 0.40562249 |
| 7 | MAPK14 | 27 | 0.04091765 | 0.37407407 |
| 8 | AKT1 | 26 | 0.06789942 | 0.39376218 |
| 9 | FYN | 25 | 0.03368482 | 0.38185255 |
| 10 | EGFR | 25 | 0.0187966 | 0.36861314 |
| 11 | ESR1 | 24 | 0.07661644 | 0.39223301 |
| 12 | VEGFA | 23 | 0.01847817 | 0.36330935 |
| 13 | LCK | 22 | 0.009664 | 0.36135957 |
| 14 | MAPK8 | 20 | 0.03380178 | 0.36594203 |
| 15 | LYN | 20 | 0.00625157 | 0.35376532 |
| 16 | AR | 18 | 0.02752194 | 0.37827715 |
| 17 | RXRA | 18 | 0.12306015 | 0.38403042 |
| 18 | NR3C1 | 18 | 0.0336209 | 0.37616387 |
| 19 | PTK2 | 17 | 0.00961775 | 0.34948097 |
| 20 | MAPK11 | 17 | 0.00943306 | 0.35314685 |

Table S4 KEGG pathway analysis of top 100 genes

| No. | Pathway name | *P* Value | Number of enriched targets |
| --- | --- | --- | --- |
| S1 | Endocrine resistance | 3.61E-21 | 22 |
| S2 | Human cytomegalovirus infection | 3.74E-20 | 28 |
| S3 | AGE-RAGE signaling pathway in diabetic complications | 1.51E-19 | 21 |
| S4 | Lipid and atherosclerosis | 1.71E-19 | 27 |
| S5 | Progesterone mediated oocyte maturation | 2.30E-19 | 21 |
| S6 | Proteoglycans in cancer | 7.72E-19 | 26 |
| S7 | Chemical carcinogenesis receptor activation | 2.46E-17 | 25 |
| S8 | Kaposi sarcoma-associated herpesvirus infection | 4.44E-17 | 24 |
| S9 | PI3K-Akt signaling pathway | 4.85E-17 | 30 |
| S10 | Prolactin signaling pathway | 8.78E-17 | 17 |
| S11 | Hepatitis B | 1.93E-16 | 22 |
| S12 | MAPK signaling pathway | 4.34E-16 | 27 |
| S13 | Relaxin signaling pathway | 5.42E-16 | 20 |
| S14 | EGFR tyrosine kinase inhibitor resistance | 6.96E-16 | 17 |
| S15 | Prostate cancer | 1.05E-15 | 18 |

Table S5 Differential metabolites in the lung of each group

| RT/min | Endogenous metabolites | Control vs. Mod | | | Mod vs. EEARH | | |
| --- | --- | --- | --- | --- | --- | --- | --- |
|  |  | *P* | VIP | Trend | *P* | VIP | Trend |
| 6.353 | Glycolic acid | 0.000032 | 1.797321 | ↓ | 0.000004 | 1.518925 | ↑ |
| 6.926 | L-Alanine | 0.000212 | 1.616759 | ↓ | 0.000042 | 1.681557 | ↑ |
| 7.333 | Glycine | 0.000356 | 1.591217 | ↓ | 0.000055 | 1.421018 | ↓ |
| 10.348 | Ethanolamine | 0.028395 | 1.141301 | ↓ | 0.003554 | 1.494809 | ↑ |
| 10.955 | L-Proline | 0.009584 | 1.297023 | ↓ | 0.002568 | 1.601473 | ↑ |
| 11.295 | Butanedioic acid | 0.000523 | 1.732494 | ↓ | 0.005562 | 1.801923 | ↑ |
| 12.239 | Serine | 0.018068 | 1.201770 | ↓ | 0.000135 | 1.747024 | ↑ |
| 13.884 | 3-Aminoisobutyric acid | 0.015295 | 1.261579 | ↑ | 0.000025 | 1.460504 | ↑ |
| 16.231 | Citrulline | 0.000153 | 1.642443 | ↑ | 0.000032 | 1.684749 | ↓ |
| 16.305 | Meso-Erythritol | 0.000224 | 1.626056 | ↓ | 0.000135 | 1.604449 | ↑ |
| 16.803 | L-5-Oxoproline | 0.041663 | 1.053770 | ↓ | 0.000248 | 1.851956 | ↑ |
| 17.091 | L-Hydroxyproline | 0.005060 | 1.360298 | ↓ | 0.000169 | 1.524165 | ↑ |
| 27.811 | D-Glucose | 0.000002 | 1.886274 | ↑ | 0.002836 | 1.217034 | ↓ |
| 28.480 | D-Mannose | 0.000020 | 1.870604 | ↑ | 0.000017 | 1.171499 | ↓ |
| 29.087 | Glycerol | 0.037163 | 1.065716 | ↓ | 0.160534 | 1.137852 | ↑ |
| 29.297 | D-Galactose | 0.000248 | 1.612840 | ↑ | 0.000003 | 1.132950 | ↓ |
| 37.904 | Naphthalene | 0.011621 | 1.283024 | ↑ | 0.000011 | 1.124738 | ↑ |
| 47.608 | Maltose | 0.026996 | 1.184361 | ↑ | 0.000005 | 1.093689 | ↑ |

Table S6 Results of pathway analysis using MetaboAnalyst database.

| No. | Pathway name | *P* Value | Match Status | Impact |
| --- | --- | --- | --- | --- |
| 1 | [Galactose metabolism](https://www.metaboanalyst.ca/MetaboAnalyst/Secure/pathway/PathResultView.xhtml) | 0.01206 | 2/27 | 0.05288 |
| 2 | [Alanine, aspartate and glutamate metabolism](https://www.metaboanalyst.ca/MetaboAnalyst/Secure/pathway/PathResultView.xhtml) | 0.01295 | 2/28 | 0 |
| 3 | [Arginine and proline metabolism](https://www.metaboanalyst.ca/MetaboAnalyst/Secure/pathway/PathResultView.xhtml) | 0.02327 | 2/38 | 0.13879 |
| 4 | [Aminoacyl-tRNA biosynthesis](https://www.metaboanalyst.ca/MetaboAnalyst/Secure/pathway/PathResultView.xhtml) | 0.03607 | 2/48 | 0 |
| 5 | [Butanoate metabolism](https://www.metaboanalyst.ca/MetaboAnalyst/Secure/pathway/PathResultView.xhtml) | 0.09292 | 1/15 | 0 |
| 6 | [Glycerolipid metabolism](https://www.metaboanalyst.ca/MetaboAnalyst/Secure/pathway/PathResultView.xhtml) | 0.09883 | 1/16 | 0.23676 |
| 7 | [Selenocompound metabolism](https://www.metaboanalyst.ca/MetaboAnalyst/Secure/pathway/PathResultView.xhtml) | 0.12213 | 1/20 | 0 |
| 8 | [Citrate cycle (TCA cycle)](https://www.metaboanalyst.ca/MetaboAnalyst/Secure/pathway/PathResultView.xhtml) | 0.12213 | 1/20 | 0.03273 |
| 9 | [Propanoate metabolism](https://www.metaboanalyst.ca/MetaboAnalyst/Secure/pathway/PathResultView.xhtml) | 0.13924 | 1/23 | 0 |
| 10 | [Glycolysis / Gluconeogenesis](https://www.metaboanalyst.ca/MetaboAnalyst/Secure/pathway/PathResultView.xhtml) | 0.15605 | 1/26 | 0.00025 |
